# Low radon exposures and lung cancer risk: joint analysis of the Czech, French, and Beaverlodge cohorts of uranium miners

**DOI:** 10.1007/s00420-019-01411-w

**Published:** 2019-02-08

**Authors:** Rachel S. D. Lane, Ladislav Tomášek, Lydia B. Zablotska, Estelle Rage, Franco Momoli, Julian Little

**Affiliations:** 1 School of Epidemiology and Public Health, Faculty of Medicine, University of Ottawa, Room 101, 600 Peter Morand Crescent, Ottawa, ON K1G 5Z3 Canada; 2 Directorate of Environmental and Radiation Protection and Assessment (DERPA), Canadian Nuclear Safety Commission (CNSC), 280 Slater Street, Station B, P.O. Box 1046, Ottawa, ON K1P 5S9 Canada; 3 National Radiation Protection Institute (SURO), Bartoskova 28, 140 00 Prague, Czech Republic; 4 Department of Epidemiology and Biostatistics, School of Medicine, University of California, San Francisco, 550 16th St, San Francisco, CA 94158 USA; 5 Institute for Radiological Protection and Nuclear Safety, B.P. 17, 92262 Fontenay-aux-Roses Cedex, France; 6 Centre for Practice-Changing Research (Room L1152), Ottawa Hospital Research Institute, Children’s Hospital of Eastern Ontario Research Institute, 401 Smyth Rd, Ottawa, ON K1H 8L1 Canada

**Keywords:** Radon, Lung cancer, Uranium mining, Epidemiology, Cohort, Risk, Smoking

## Abstract

It is well established that high radon exposures increase the risk of lung cancer mortality. The effects of low occupational exposures and the factors that confound and modify this risk are not clear and are needed to inform current radiation protection of miners. The risk of lung cancer mortality at low radon exposures (< 100 working-level months) was assessed in the joint cohort analysis of Czech, French, and Canadian uranium miners, employed in 1953 or later. Statistical analysis was based on linear Poisson regression modeling with grouped cohort survival data. Two sensitivity analyses were used to assess potential confounding from tobacco smoking. A statistically significant linear relationship between radon exposure and lung cancer mortality was found. The excess relative risk per working-level month was 0.022 (95% confidence intervals: 0.013–0.034), based on 408 lung cancer deaths and 394,236 person-years of risk. Time since exposure was a statistically significant modifier; risk decreased with increasing time since exposure. A tendency for a decrease in risk with increasing attained age was observed, but this was not statistically significant. Exposure rate was not found to be a modifier of the excess relative risk. The potential confounding effect of tobacco smoking was estimated to be small and did not substantially change the radon–lung cancer mortality risk estimates. This joint cohort analysis provides strong evidence for an increased risk of lung cancer mortality from low occupational radon exposures. The results suggest that radiation protection measures continue to be important among current uranium miners.

**Electronic supplementary material:**

The online version of this article (10.1007/s00420-019-01411-w) contains supplementary material, which is available to authorized users.

## Introduction

It is well established that in underground miners with very high radon exposures, there is a linear dose–response relationship between radon exposure and lung cancer mortality (National Research Council 1999). This is the primary basis for the classification of radon (^222^Rn) and its short lived progeny (^218^ Po, ^214^Pb, ^214^Bi, and ^214^Po) as Group 1 human carcinogens by the International Agency for Research on Cancer (IARC) (IARC 2012b). The term radon is used throughout this article and refers to radon and radon progeny, synonymously.

Evidence of the radon–lung cancer relationship derives largely from studies of miners who started working prior to time periods when radiation protection measures substantially reduced radon exposures in mines and when exposures were largely based on imprecise estimates. The long-term lung cancer risk of current miners exposed to very low radon exposures [mean < 0.25 working-level month per year (WLM/year)] is less certain. It is not feasible to conduct long-term follow-up cohort studies at such low exposures, especially because of the lack of statistical power and the importance of the confounding effects of tobacco smoking and residential radon exposure (CNSC 2003). Relevant evidence can be drawn from updated historic cohorts of uranium miners, particularly during periods after radon mitigation measures were in place and exposure measurements were of high quality.

Studies of underground miners, including joint cohort analyses (National Research Council 1999; Tomasek et al. 2008; Lubin et al. 1997), updated cohorts (Tomasek 2012; Lane et al. 2010; Rage et al. 2015, 2018; Walsh et al. 2015; Navaranjan et al. 2010, 2016; Kreuzer et al. 2015, 2018; Vacquier et al. 2009), and nested case–control studies (Hunter et al. 2013; Leuraud et al. 2011), have advanced our understanding of the health effects of radon. Several analyses have focused on relatively low radon exposures and exposure rates, or time periods during which routine radon measurements in work areas and/or of individuals were made, as distinct from estimates or extrapolations (Tomasek et al. 2008; Lubin et al. 1997; Rage et al. 2012, 2015, 2018; Navaranjan et al. 2016; Kreuzer et al. 2015, 2018; Hunter et al. 2013; Leuraud et al. 2011; Vacquier et al. 2011). These studies are important, since they provide statistically significant lung cancer estimates at occupational radon exposure of about 50 WLM. However, individual cohort studies have limited statistical power to assess lung cancer risk at low exposures. Likewise, the past joint cohort studies included time periods in which radon exposure was estimated or extrapolated. In the assessment of risk associated with low radon exposures, addressing sources of potential confounding is of concern. In particular, tobacco smoking is the primary cause of lung cancer worldwide (IARC 2012c). Uranium miners are known to have high prevalence rates of tobacco smoking (National Research Council 1999; Kreuzer et al. 2018; Hunter et al. 2013; Leuraud et al. 2007, 2011; Villeneuve et al. 2007; Schubauer-Berigan et al. 2009; Schnelzer et al. 2010; Tomasek 2011, 2013; L’Abbé et al. 1991; Amabile et al. 2009). A challenge in historic cohort studies is that information on tobacco smoking is not readily available. However, some cohorts have smoking information for a subset of miners (Kreuzer et al. 2015, 2018; Villeneuve et al. 2007; Schubauer-Berigan et al. 2009) and some have smoking information from case–control studies nested within them (Hunter et al. 2013; Leuraud et al. 2007, 2011; Schnelzer et al. 2010; Tomasek 2011, 2013; L’Abbé et al. 1991; Amabile et al. 2009).

The French and Czech cohorts of uranium miners had been combined for time periods when radiation protection and routine radon monitoring programs were in place (Tomasek et al. 2008). The Beaverlodge cohort of uranium miners, which was part of the Canadian Eldorado cohort, included a large number of workers employed after routine radiation protection and regular radon monitoring were in place (Lane et al. 2010). Miners from these three cohorts can be used to estimate the radon–lung cancer risk of current uranium miners. Therefore, this paper reports on the joint analysis of the French, Czech, and Beaverlodge cohort of uranium miners. These miners were employed after radon mitigation measures were in place and when individual radon exposure data were of good quality. The main objective of this study was to assess the radon–lung cancer mortality relationship at low radon exposures (< 100 WLM) and to consider carefully the possible impact of tobacco smoking, and modifying factors, on this relationship.

## Methods

### Study design and grouped person-year data

This study is based on three historic cohorts of uranium miners from the Czech Republic, France, and Canada. Their characteristics (i.e., type of workers, mine location, and time period of employment) are summarized in Table 1. Detailed information is reported elsewhere for the Czech (Tomasek et al. 2008), French (Vacquier et al. 2009) and Beaverlodge (Lane et al. 2010) cohorts.

**Table 1:** Characteristics of the historic Czech, French, and Canadian (Beaverlodge) cohorts of uranium miners

Cohort	Type of worker	Mine location	Time period of employment	Inclusion criteria	Person-years at risk
Czech (Tomasek et al. 2008)	Underground miners	Jáchymov region of West Bohemia	1948–1959	Employed at least 4 years	From the earliest year of follow-up plus 4 years until the end of follow-up
		Příbram district of Central Bohemia	1968–1988	Employed at least 1 year	From the earliest year of follow-up plus 1 year until the end of follow-up
French (Vacquier et al. 2009)	Underground and open pit miners	Four main mining areas in France: Vendée, Limousin, Forez and Hérault	1946–1999	Employed at least 1 year	From the earliest year of follow-up plus 1 year until the end of follow-up
Beaverlodge, Canada (Lane et al. 2010)	Underground miners and mill workers	Near town of Eldorado in Northern Saskatchewan	1948–June 1982 Workers employed in 1982 had exposure follow-up to 1999	Employed some time from 1948 to 1980, were aged 15–75 years when employed, and known to be alive in 1950 at the start of the mortality follow-up	From the date of the first day of employment until the end of follow-up

Restricted calendar year ranges were established for each cohort to harmonize the three cohorts as much as possible in terms of the nature and measurement of exposure for the current analysis (Table 2). Thus, the cohorts were restricted to time periods after radon protection measures were introduced, especially mechanical ventilation systems, and when radon progeny measurements were routinely made, in work areas and/or of individuals, as part of regulatory requirements. These periods correspond to lower radon exposures and lower radon exposure rates (Tomasek et al. 2008; Lane et al. 2010; Vacquier et al. 2011; Rage et al. 2012). Thus, 1953–1999, 1956–1999, and 1965–1999 were the time periods used for the Czech, French, and Beaverlodge cohorts of uranium miners, respectively, for this analysis.

**Table 2:** Harmonizing the cohorts by time periods of radiation protection measures and quality of radon exposure assessment for the current analysis

Cohort	Start of follow-up	Start of routine radon monitoring	Start of mechanical ventilation	Mean annual radon exposures
Czech (Tomasek et al. 2008)	1953 A priori	1949 Extensive radon gas measurements were conducted since 1949 in nearly all shafts; about 200 measurements per shaft and year from 1949 to 1960 and more than 900 per shaft per year afterwards	1953	Decreased from 25 to 7 WLM/year from 1952 to 1967; about 1 WLM/year thereafter
French (Tomasek et al. 2008; Vacquier et al. 2011; Rage et al. 2012)	1956 A priori	1956 From 1956 to 1982, individual exposure was assessed from ambient measures and characteristics of job (job type & time spent in the mine); individual exposure was measured with individual dosimeters from 1983	1956	Decreased from > 20 WLM/year from 1946 to 1955 to > 4 WLM/year thereafter
Beaverlodge (Lane et al. 2010)	1965	1966 The total number of radon measurements taken per workplace per year from 1954 to 1968 were generally less than 12, with an average of about 4 measurements per workplace per year. Personal exposures were first assigned to underground uranium miners in November 1966	1952	Decreased from 50 WLM/year in the 1950s to 20 WLM/year in the 1960s and to < 3 WLM/year from the 1970s onward

Unfortunately, because of privacy restrictions, only the aggregate person-year tables were available for the Beaverlodge cohort. Thus, person-year tables were provided specific for this analysis for the Czech and French cohorts. No individual information was available.

For all workers, person-years were accumulated from the date of entry into the cohort (the start of follow-up). This was the date of first employment plus 1 year for the French (i.e., 1956 + 1 year) and Czech (Příbram) cohort (i.e., 1968 + 1 year), the date of first employment plus 4 years for the Czech (Jáchymov) cohort (i.e., 1953 + 4 years), and the first day of employment for the Beaverlodge workers (i.e., 1965). In the Czech and French cohorts, each individual contributed person-years from the start of follow-up to the earliest of the date of death, emigration (Czech), loss to follow-up, age 85 years, or December 31, 1999, which was the end of the study period (Tomasek et al. 2008; Vacquier et al. 2011; Rage et al. 2012). In the Beaverlodge cohort, each individual contributed person-years from the start of follow-up to the earliest of the date of death, the last date known alive, or December 31, 1999 (Lane et al. 2010). For comparability reasons, the end of the study period was 1999 for all cohorts [despite the French and Czech cohorts having been updated to 2007 (Rage et al. 2015, 2018) and 2010 (Tomasek 2012), respectively].

Beaverlodge person-time contributions before 1965 were excluded for this analysis; however, post-1965 person-years were included among workers first hired before 1965 who continued to work after 1965. This could introduce a potential healthy worker survivor selection bias, by mixing of “prevalent” and “incident” hires (Costello et al. 2011; Applebaum et al. 2007).

Two sensitivity analyses assessed the impact of “prevalent” hires in the Beaverlodge cohort. The first sensitivity analysis compared the excess relative risk/working-level month (ERR/WLM) of Beaverlodge person-year restrictions to calendar year periods 1950–1999 (incident hires) and 1965–1999 (includes prevalent hires). In the second sensitivity analysis, it was not possible to determine how many workers started working before 1965 and continued working after 1965; however, the number of person-years before and after 1965 was determined.

The Czech and French cohorts had a minimum 1-year or 4-year employment eligibility criterion. The Beaverlodge cohort did not restrict workers by duration of employment. All workers on the payroll for 1 day or more were included; however, the summary person-year experience was cross-classified by total duration of employment (< 6 months and ≥ 6 months). This was because lung cancer risk was high before 6 months, decreased after 6 months then remained constant (Lane et al. 2010). Person-years for those employed < 6 months were excluded from the current joint analysis to reduce potential healthy worker survivor bias (Buckley et al. 2015) and to better align Beaverlodge with the Czech and French miners, who had a minimum 1-year or 4-year employment eligibility criterion. The person-year tables would not allow us to perform sensitivity analysis with a minimum of 1 year to be homogeneous with the Czech and French cohorts. Beaverlodge workers employed < 6 months represented 27.5% (74) of lung cancer deaths and 39.3% (97,617) of person-years at risk from 1965 to 1999. These workers were younger (22.7% of person-years at ages 16–29 years versus 4.8%), had lower radon exposures (87.9% of person-years at risk < 5 WLM versus 45.3%) and lower mean radon exposures (9.97 WLM; SD 15.4; max. 9.5 WLM versus 118.5 WLM; SD 195.8; max. 1617) compared to workers employed ≥ 6 months.

### Lung cancer mortality and follow-up

The International Classification of Disease (ICD) codes (WHO 1998) were used to code and classify mortality data from vital statistics sources (Table 3). The original ICD codes for underlying cause of death of lung cancer were recoded to the ICD 9th revision.

**Table 3:** Sources of lung cancer outcome information

Cohort	Vital statistics	Cause of death	Proportion of missing causes of death	Histological confirmation of lung cancer?
Czech (Tomasek et al. 2008)	Czech Population Registry at the Ministry of the Interior	Before 1982 from local death registries. After 1982 from the Institute of Health, Information and Statistics of the Czech Republic	About 0.4%	Histological confirmation on about 80% of the lung cancers
France (Vacquier et al. 2011; Rage et al. 2012)	National Directory for the Identification of Natural Persons/RNIPP	Before 1968 from administrative and medical files of Compagnie Générales des Matières Nucléaires Inc. (COGEMA). After 1968 from a standardized anonymous linkage to the French National Epidemiological Centre on Medical Causes of Death, supplemented by COGEMA medical files	About 32% before 1965; 28% from 1965 to 1969 (prior to the national mortality database); 0.8% thereafter	No histology
Beaverlodge (Lane et al. 2010)	Deaths are registered by the province/territory, where the event occurred and are sent to Statistics Canada	1950–1999 from the Canadian national mortality database. This database contains records of all deaths registered in Canada and voluntarily reported deaths of Canadian residents occurring in the United States	About 1% or less	Lung cancer histology was available for workers with lung cancer incidence records

The person-years for the French cohort were corrected to adjust for 32% missing causes of death in France before 1965, and 28% missing causes of death from 1965 to 1969 prior to when the national mortality database existed, as the rates in early years would be otherwise underestimated. Only 0.8% of deaths had missing causes, thereafter (Tomasek et al. 2008).

In the Czech and French cohorts, follow-up was censored at age 85 years to reduce potential bias due to missing information and uncertainty of diagnoses in older age categories (Tomasek et al. 2008); a relatively high proportion of causes of death in older workers were submitted by doctors (not based on autopsy) and are known to be subject to error (Rage et al. 2012). In the Beaverlodge cohort, follow-up was censored at age 100 years (Lane et al. 2010). Misclassification of lung cancer deaths from Beaverlodge workers age 85–100 years would have minimal impact on risk estimates because of the high quality of Canadian cancer incidence and mortality databases (Statistics Canada 2013).

Only 2.5% of miners were lost to follow-up, based on the joint analysis of the Czech and French cohorts that this study is largely based (Tomasek et al. 2008). Less than 1% of miners were lost to follow-up in the Alpha Risk project (Tirmarche et al. 2010) among Czech (1948–1999) and French (1946–1999) cohorts; likewise, about 1% of French miners (1956–1999) were lost to follow-up (Vacquier et al. 2011). In the Eldorado cohort, 7% of workers with a social insurance number (SIN) were lost to follow-up (CNSC 2004); however, this information was not provided for the Eldorado sub-cohorts (Lane et al. 2010). Most Beaverlodge workers first employed from 1965 would have a SIN; this would substantially improve record linkage. Loss to follow-up of Beaverlodge workers in this analysis is most likely less than 7%.

### Exposure: radon progeny

Radon progeny exposure was measured during monitoring activities, and reported as radon gas concentrations (converted to radon progeny concentrations using equilibrium factors in mines) or direct radon progeny concentrations in ambient air and characterized with employment details (e.g., duration of underground work in different shafts or other work areas within mines, and job type). Alternatively, personal alpha dosimeters (PADs), worn by miners, determined the potential alpha-particle energy of radon progeny to the individual. Radon progeny exposures were considered high quality if a large number of ambient radon gas or radon progeny measurements taken in specific work areas within the mines, or individual radon progeny exposures determined directly from PADs, were taken during the follow-up periods (Table 2). Details of the exposure measurement methods are reported elsewhere (Tomasek et al. 2008; Lane et al. 2010; Vacquier et al. 2011; Rage et al. 2012).

Occupational exposure to radon and its progeny was characterized as the product of time in the workplace (months) and the radon progeny concentration, measured in working levels (WL) in the workplace air, resulting in working-level months (WLM). One WL is the concentration of radon progeny in 1 L of air that will result in the emission of 1.3 × 10^5^ MeV of potential alpha-particle energy after complete decay. Radon progeny concentrations (WL) were converted to radon progeny exposures by multiplying by 170 h worked (1 month of work corresponds to 170 h). Therefore, one WLM is the cumulative exposure to an individual from breathing in an atmosphere, at a radon progeny concentration of 1 WL, over one working month of 170 h (ICRP 2010). In the present paper, radon exposure is expressed as a cumulative mean weighted by person-years, and lagged by 5 years, throughout follow-up.

### Statistical analysis

The primary statistical analysis was based on an internal Poisson regression model with grouped survival data (Breslow and Day 1987; Preston and Users Group (SESUG) 1996; Preston and Shilnikova 2017). Person-year grouped data were cross-classified on several time scales and other relevant explanatory variables. This included three sub-cohorts (Czech, French, and Beaverlodge) and standardized strata: 12 strata of age at risk (≤ 29; 5 year intervals from 30 to 34 to 75–79; 80–100), and 9 strata of calendar year at risk (5 year intervals from 1955–1959 to 1995–1999). Each stratum was further described by mean radon exposure (i.e., 5-year lagged and weighted by person-years at risk), mean age at first radon exposure, mean time since first exposure, and mean exposure rate.

Radon exposures were lagged by 5 years to account for a minimal induction time and to control for issues of ‘reverse causality’ (Buckley et al. 2015). A 5-year lag interval has a strong theoretical base (National Research Council 1999; IARC 2012a) and has been used in most analyses of cancer risk in uranium miners (Tomasek 2012; Lane et al. 2010; Navaranjan et al. 2016; Kreuzer et al. 2015, 2018; Rage et al. 2018).

Linear excess relative risk (ERR) models were fit using background stratification to estimate background rates (National Research Council 1999; Richardson et al. 2012), which depend mostly on age and sub-cohort:

1 $${Rate_{w}}~=~{Rate_{o}}~ \times ~(1.0~+~\beta w),$$

where Rate_*w*_ is the lung cancer mortality rate at cumulative exposure *w*, and w is the 5-year lagged continuous exposure, in WLM. Rate_*o*_ is the background lung cancer mortality rate, stratified for three strata of sub-cohort and 12 strata of age at risk. Calendar year at risk is closely associated with average exposure rate, duration of exposure, time since exposure (National Research Council 1999), and especially the quality of radon exposure measurements. Thus, the background lung cancer mortality rate was not stratified by calendar year at risk for this analysis to avoid collinearity with these factors. In the model, *β* estimates the ERR per unit of radon exposure in WLM. Adding 1.0 to the ERR/WLM results in the Relative Risk (RR) at 100 WLM of radon exposure.

Most analyses were restricted to cumulative radon exposures < 100 WLM for the three cohorts separately and combined. The reference category represented hypothetical workers at 0.0 WLM when the results are presented as rate ratios or relative risks (RR) in different exposure categories. Exposure categories are based on a relatively even distribution of the number of lung cancer deaths.

We also addressed effect modification, by expanding Eq. 1 to assess whether the radon–lung cancer mortality relationship depended on three time since exposure windows (5–14, 15–24, and 25 + years), four attained age categories (< 55, 55–64, 65–74, and 75 + years), or three or six exposure rate categories (< 0.5, 0.5–4.9, 5.0 + WL; < 0.5, 0.5–1.0, 1.0–3.0, 3.0–5.0, 5.0–15, and 15 + WL). Covariate adjustment (or modifying effects of other variables) is used for better fit, as the linear model (more precisely its regression coefficient) is not the same for all other variables (such as time, age, and exposure rate).

A modified ERR model was used, such that

2 $${Rate_{w}}~=~{Rate_{o}}~ \times \left[ {1.0~+(\beta w)~\exp ~\left( {{{\mathop \sum \nolimits^{} }_i}{\gamma _i}~{z_i}} \right)} \right],$$

where *z*_*i*_ are potential modifying factors and exponentiated *γ*_*i*_ are the estimated RRs of the modifying factors relative to a baseline ERR.

Two sensitivity analyses assessed the consistency of the effect modifier findings. The first combined two of each of the three sub-cohorts and the second limited the initial joint cohort to exposure rates ≤ 5.0 WL (616 lung cancer deaths and 419,521 person-years of follow-up).

All statistical analyses used the AMFIT module in the EPIWIN implementation of EPICURE for Windows (Preston and Shilnikova 2017). Tests of statistical significance used the likelihood ratio test, comparing two nested models with and without the radon exposure variable. A restriction was imposed on possible values of *β* from the ERR models, such that the corresponding RR estimate could not be negative (Zablotska et al. 2013).

### Potential confounding by unmeasured tobacco smoking

The prevalence of tobacco smoking was not available in the Czech, French, or Beaverlodge cohorts of uranium miners used in this analysis. Two different sensitivity analyses were devised to provide a range of possible indirect estimates of the magnitude of the confounding effect of tobacco smoking had it been possible to control for it directly. The first method (Steenland and Greenland 2004) relied on external information on the smoking prevalence among miners, and external information on the strength of association of lung cancer mortality for current (current or ex-smoker < 10 years), former (ex-smoker ≥ 10 years), and never smokers (Hunter et al. 2013). External information on the smoking prevalence of uranium miners for radon-exposed workers (50–99 WLM) and workers at reduced exposure (a reference level of < 25 WLM) came from special tabulations of the European joint nested case–control study of uranium miners (Hunter et al. 2013) (Supplementary Table 1; Hunter, special tabulations, 2016-10-17). External information on the strength of association of lung cancer mortality for current (RR = 8.96) and former (RR 3.85) smokers compared to never smokers came from a meta-analysis of tobacco smoking and cancer that included the tobacco–lung cancer relationship (Gandini et al. 2008).

The prevalence of ever-smokers was also adjusted to reflect the range of reported smoking prevalence rates (60–80%) among uranium miners (CNSC 2003; Kreuzer et al. 2018; Villeneuve et al. 2007; Tomasek 2011, 2013; L’Abbé et al. 1991; Leuraud et al. 2007; Amabile et al. 2009). If *p*(never, 50–99 WLM), *p*(former, 50–99 WLM), *p*(current, 50–99 WLM) and *p*(never, < 25 WLM), *p*(former, < 25 WLM), *p*(current, < 25 WLM) are the proportions of never, former, and current smokers in the two categories of radon exposure, then the elevation in the rate of lung cancer mortality due to tobacco smoking would be a weighted average of the smoking-specific rate ratios (Steenland and Greenland 2004).

To derive the magnitude of confounding bias due to unmeasured smoking, we assumed that there were no other unmeasured confounders, radon exposure had no effect on the risk of lung cancer mortality, and tobacco smoking was not an effect modifier of the radon–lung cancer relationship. Thus, the magnitude of bias in the comparison of radon-exposed miners to reference level miners would be derived by

3 $${\text{Bias}}=~\frac{{R{R_{50-99\;{\text{WLM}}}}~}}{{~R{R_{<\,25\;{\text{WLM}}}}}}=\frac{{{p_{\text{never},~50-99~\;{\text{WLM}}}}+~\exp ~({\beta _2})~{p_{{\text{former}},~50-99\;{\text{WLM}}}}~~+~\exp ~({\beta _3})~{p_{{\text{current}},~~50-99\;{\text{WLM}}}}~}}{{{p_{{\text{never}},~<\,25\;{\text{WLM}}}}+~\exp ~({\beta _2})~{p_{{\text{former}},~<~25\;{\text{WLM}}}}~~+~\exp ~({\beta _3})~{p_{{\text{current}},~~<~25\;{\text{WLM}}}}~}}.$$

The smoking-adjusted RR for radon exposure can then be estimated by dividing the unadjusted RR derived in the primary analyses by the bias factor estimated above:

4 $${\text{R}}{{\text{R}}_{{\text{adj}}}}=~\frac{{{\text{R}}{{\text{R}}_{{\text{unadj}}}}}}{{{\text{Bias}}}}.$$

The second method relied on the estimated magnitude of bias from a direct comparison of smoking-unadjusted RR (range of 1.25–4.90) and smoking-adjusted RRs (range of 1.23–4.93) of lung cancer mortality at 100 WLM. This was based on findings from nested case–control studies and the 1960 + sub-cohort of German uranium miners, in which smoking status is available for 56% cohort members (Supplementary Table 2) (Kreuzer et al. 2018; Hunter et al. 2013; Leuraud et al. 2007, 2011; Schnelzer et al. 2010; Tomasek 2011, 2013; L’Abbé et al. 1991). The bias factor was calculated by dividing the smoking-unadjusted RR by the smoking-adjusted RR. A range of smoking-adjusted RRs in the primary data set was then estimated by dividing the smoking-unadjusted RR in the primary analysis by each bias factor, in turn (range of 0.99–1.22).

### Ethical issues

The Ottawa Health Sciences Network—Research Ethics Board approved the research for this study (Ref #20150478-01H).

## Results

The study’s main objective was to assess the radon–lung cancer mortality relationship of low radon exposures; thus, the joint cohort (< 100 WLM) represented 62.9% (408) of the lung cancer deaths and 92.0% (394,236) of person-years at risk with a mean cumulative radon exposure (5-year lagged, WLM; weighted by person-years at risk) of 36.42 WLM, compared to the initial joint cohort (649 lung cancer deaths; 428,356 person-years; mean 95.49 WLM). Table 4  describes the characteristics of the Czech, French, and Beaverlodge cohorts, respectively.

**Table 4:** Description of cohorts

Characteristics	Czech (1953–1999)	French (1956–1999)	Beaverlodge (1965–1999)
Total			
Lung cancer deaths	389	65	195
Person-years	196,533	80,859	150,964
Person-years at 0.0 WLM^a^	28,360	25,384	18,711
Mean (WLM)^b^	95.5	36.4	118.5
Range (WLM)	0–363	0–127.7	0–1,617
Mean exposure rate (WL)	1.02	0.27	2.64
Range (WL)	0–5.10	0–7.04	0–29.44
< 100 WLM			
Lung cancer deaths	223	62	123
Person-years	179,837	80,286	134,113
Mean (WLM)^b^	45.1	32.9	32.3
Mean exposure rate (WL)	0.67	0.27	1.45
Range (WL)	0–5.10	0–7.04	0–17.58

^a^Individual number of workers with 0.0 WLM not available, only person-year tables are provided

^b^Mean cumulative radon exposure (5-year lagged, WLM) weighted by person-years at risk

### Exposure response analyses

A statistically significant excess relative risk of lung cancer mortality was observed for the joint cohort (ERR/WLM = 0.022; 95% CI 0.013–0.034, *P* < 0.001). The French and Beaverlodge cohorts had the lowest and highest risk estimates (ERR/WLM = 0.020 and 0.024, respectively); all three cohort’s 95% confidence intervals included the other cohort’s risk estimates. An ERR/WLM of 0.029 (95% CI 0.014–0.050; *P* < 0.001) was found for the joint cohort restricted to < 50 WLM (276 lung cancer deaths, 360,370 person-years at risk, mean = 20.35 WLM) (Table 5).

**Table 5:** Excess relative risks of lung cancer mortality by mean radon exposure, joint cohort < 100 WLM

Cohort	Lung cancer deaths	Person-years	Mean cumulative^a^ exposure (WLM)	ERR/WLM^b^ (MLE)	95% CI^c^
Joint	408	394,236	36.42	0.022	0.013–0.034
Czech	223	179,837	45.1	0.021	0.010–0.040
French	62	80,286	32.9	0.020	0.005–0.051
Beaverlodge	123	134,113	32.3	0.024	0.009–0.047

*WLM* working-level months, *ERR/WLM* excess relative risk per working-level month, *CI* confidence interval, *MLE* maximum likelihood estimate

^a^Mean cumulative radon exposure (5-year lagged, WLM) weighted by person-years at risk

^b^Poisson regression model, for grouped survival data, with background stratification by sub-cohort (3 categories) and age at risk (12 categories)

^c^Likelihood bounds for exposure variable (WLM)

**Fig. 1:**
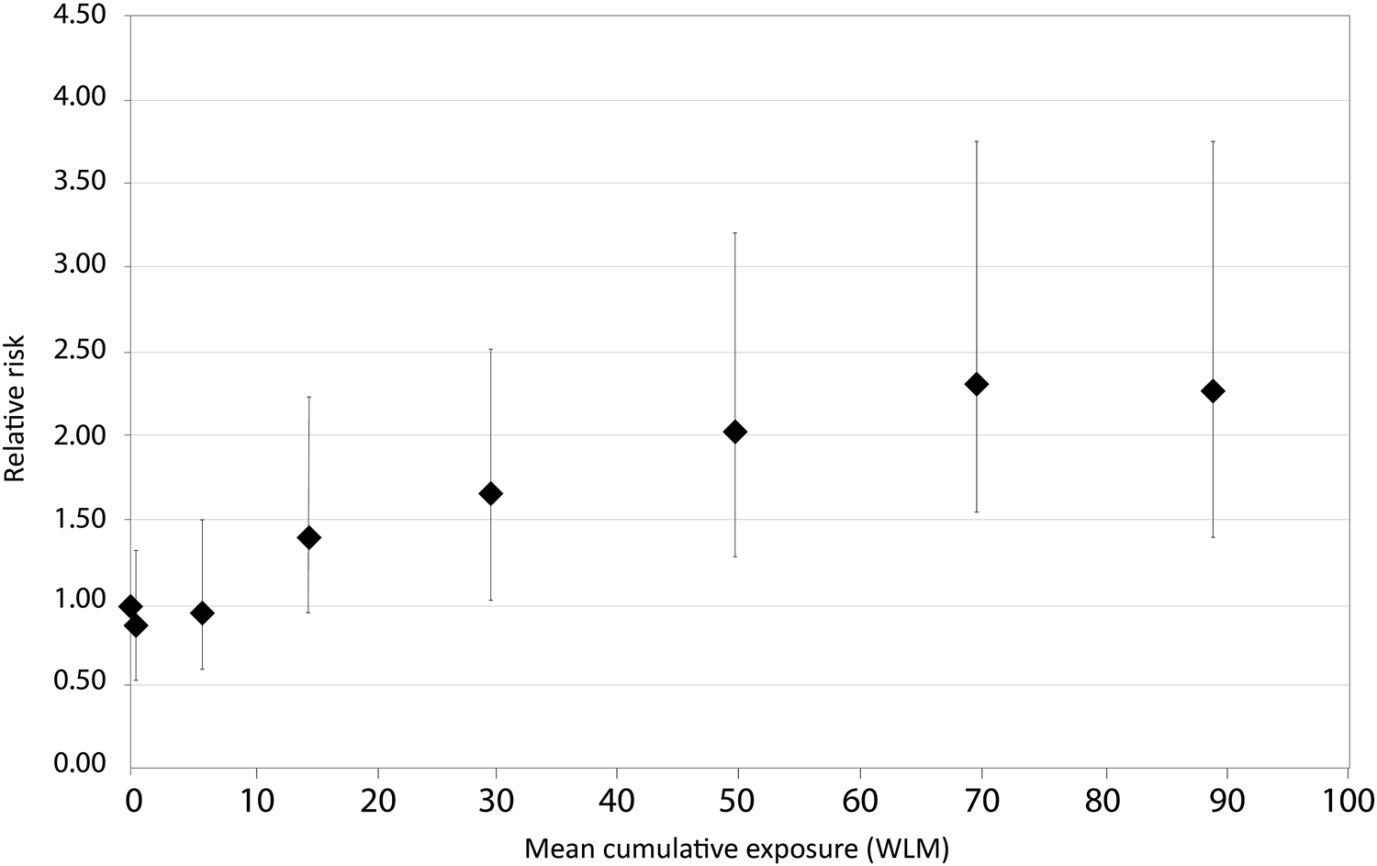
Relative risk of lung cancer mortality by categories of exposure < 100 WLM for the joint cohort, 1953–1999

The secondary, categorical analysis of the joint cohort (Fig. 1; Table 6) had a monotonic increase in relative risk. The linear trend was statistically significant (*P* < 0.001).

**Table 6:** Relative risks of lung cancer mortality by categories of radon exposure, joint cohort restricted to < 100 WLM

Cumulative radon exposure (WLM)	Mean cumulative exposure (WLM)^a^	Lung cancer deaths	Person-years	Relative risk (MLE)	95% CI	LR statistic	Degrees of freedom	*P* Value (Linear trend)
0.0	0.0	32	72,455	1.00		51.08	7	< 0.001
> 0.0–2	1.5	39	109,620	0.83	0.51–1.34			
3–9	6.0	51	84,256	0.94	0.60–1.50			
10–19	14.6	48	44,444	1.41	0.90–2.25			
20–39	29.6	63	34,915	1.62	1.06–2.52			
40–59	49.5	66	21,157	2.02	1.31–3.18			
60–79	69.4	64	17,147	2.39	1.55–3.76			
80–100	88.7	45	10,244	2.32	1.45–3.76			
Total		408	394,236					

Deviance 2607.234

Relative risks were stratified by sub-cohort (3 categories) and age at risk (12 categories)

*P* value of the test of linear trend is based on mean values for exposure categories

Workers with zero cumulative radon exposures are included in the 0.0 WLM category; workers who had cumulative exposures ranging from 0.010 to 2.999 WLM are included in the > 0.0–2 WLM category

*WLM* working-level months, *CI* confidence interval, *MLE* maximum likelihood estimate, *LR* likelihood ratio

^a^Mean cumulative radon exposure (5-year lagged, WLM) weighted by person-years at risk

The first sensitivity analysis to compare the ERR/WLM of Beaverlodge restrictions to calendar years 1950–1999 (incident hires) and 1965–1999 (prevalent hires) found minimal difference between the two calendar time periods for the whole range of cumulative radon exposures. The total Beaverlodge cohort (1950–1999) had 279 lung cancer deaths, 285,846 person-years, and ERR/WLM = 0.0092. The restricted Beaverlodge cohort for 1965–1999 had 269 lung cancer deaths, 248,580 person-years, and ERR/WLM = 0.0091.

For the second sensitivity analysis, there were few person-years in 1965–1970 with radon exposures received over 10 years prior (~ 7000 person-years), but a sizable proportion were received up to 5 years prior (~ 22,000 person-years).

### Effect modifiers

The ERR/WLM of 0.022 (95% CI 0.013–0.034, *P* < 0.001) is the ERR/WLM for the reference category of the effect modifier analysis (< 100 WLM joint cohort, sub-cohort, and age at risk were stratifying variables). The ERR/WLM decreased significantly with increasing time since exposure. Exposures received 5–14 years previously were ERR/WLM = 0.035; 95% CI 0.018–0.060; the corresponding risk at least 25 + years was about one-third the risk (ERR/WLM = 0.012; 95% CI 0.004–0.023). Only time since exposure led to a statistically significant improved fit (LR *P* value = 0.002) over the initial model (Table 7).

**Table 7:** Excess relative risk and relative risk estimates of lung cancer mortality from the interaction models, joint cohort < 100 WLM

Parameter	Deaths	Model A		Model B		Model C		Model D	
		Parameter estimate^a^	95% CI	Parameter estimate^a^	95% CI	Parameter estimate^a^	95% CI	Parameter estimate^a^	95% CI
Background									
(*β*) ERR/WLM	408	0.022	0.013–0.034	0.035	0.018–0.060	0.047	0.020–0.096	0.056	0.020–0.133

				Relative risk^b^	95% CI	Relative risk^b^	95% CI	Relative risk^b^	95% CI
Effect modifiers									
Time since exposure (WLM)									
5–14 years				1.00		1.00		1.00	
15–24 years				0.96	0.50–1.90	0.96	0.50–1.86	0.89	0.45–1.74
25 + years				0.33	0.12–0.70	0.36	0.11–0.76	0.38	0.14–0.78
Attained age (years)									
< 55	119					1.00		1.00	
55–64	167					0.82	0.30–2.17	0.78	0.29–2.10
65–74	99					0.37	0.02–1.35	0.37	0.04–1.26
75 +	23					0.26	0.00–3.24	0.02	N/A
Exposure rate (WL)									
< 0.5	212							1.00	
0.5–4.9	182							0.83	0.48–1.66
5.0 +	14							2.00	0.44–5.86

Maximum likelihood estimates and 95% confidence interval

*WL* working levels, *WLM* working-level months, *ERR/WLM* excess relative risk per working-level months, *CI* confidence interval, *N/A* not available due to convergence problem in the EPICURE package

^a^ERR/WLM for cumulative radon exposure, 5-year lagged

^b^Relative risks for time since exposure, attained age, and exposure rate variables. Stratification was by sub-cohort (3 categories), and age at risk (12 categories). Parameters are estimated on the basis of the model below. Here the bracketed area represents the cumulative exposures and parameter estimates obtained in different time windows (5–14, 15–24, and 25 + years previously). Subscript *a* denotes categories of attained age and the subscript *z* denotes categories of radon concentration in WL

$${\text{Rat}}{{\text{e}}_w}=\left[ {1.0~+~\beta ~\left( {~{w_{5 - 14}}+{\theta _{15 - 24}}{w_{15 - 24}}~+~{\theta _{25+}}{w_{25+}}~} \right) \times {\gamma _a}~{z_z}} \right]$$

Similar to the primary analyses, both sensitivity analyses [the first combined two of each of the three cohorts and the second limited the initial joint cohort to exposure rates ≤ 5.0 WL] found statistically significant ERR/WLM of lung cancer mortality with increasing radon exposure. The ERR/WLM at exposure rates ≤ 5.0 WL was 0.023 (95% CI 0.017–0.032, *P* < 0.001), based on 616 lung cancer deaths, without taking effect modifiers into account. The excess relative risk decreased significantly with increasing time since exposure; for exposures received 5–14 years previously the ERR/WLM = 0.043; 95% CI 0.030–0.061; the corresponding risk at least 25 + years was again about one-third the risk (0.043 × 0.31 = 0.013) and led to a statistically significant improved fit (LR *P* value < 0.001) over the initial model. A decrease in risk with increasing attained age was also observed (LR *P* value = 0.016).

### Potential confounding by unmeasured tobacco smoking

Table 8 gives the smoking-unadjusted RRs for the joint cohort analysis. Cumulative radon exposure categories were simplified to correspond to the external European joint nested case–control study (Supplementary Table 1) (Hunter et al. 2013) which provided the smoking prevalence among exposed (50–99 WLM) and reference exposed (< 25 WLM) uranium miners (Nezahat Hunter, special tabulations, 2016-10-17).

**Table 8:** Smoking-unadjusted relative risks of lung cancer by radon exposure (WLM), joint cohort < 100 WLM

Exposure (WLM)	Lung cancer deaths	Person-years	Relative risk No adjustment for smoking
< 25	192	325,049	1.00
25–49	84	35,321	1.85
50–99	132	33,865	2.07
Total	408	394,236	

The reference category (< 25 WLM) has a relative risk set to unity. Smoking-unadjusted RRs for the primary data set for analysis (i.e., joint cohort study) < 100 WLM and are stratified by sub-cohort (3 categories) and age at risk (12 categories)

Mean cumulative radon exposure (5-year lagged, WLM) weighted by person-years at risk

The first external sensitivity analysis, using the European smoking prevalence data and external risk estimates of smokers’ lung cancer mortality (Gandini et al. 2008), resulted in a bias factor of 0.916 and a smoking-adjusted RR of 2.26 (Eq. 4) for ever-smoking exposed miners (50–99 WLM). When we varied the range of smoking prevalence of ever-smokers (60–80%) in 30 different scenarios, a range of bias factors (0.89–1.10) (not shown) and smoking-adjusted risk estimates (RR 1.88–2.33) for ever-smoking exposed miners (50–99 WLM) was found.

The second external sensitivity analysis used published nested case–control studies of tobacco smoking and the 1960 + sub-cohort of German uranium miners (Supplementary Table 2) (Kreuzer et al. 2018; Hunter et al. 2013; Leuraud et al. 2007, 2011; Schnelzer et al. 2010; Tomasek 2011, 2013; L’Abbé et al. 1991) to calculate a range of bias factors. The smoking-unadjusted RR was 3.2 at 100 WLM (ERR/WLM = 0.022) from the primary analysis (Table 5). Thus, the resulting smoking-adjusted RRs ranged from 2.62 to 3.23 at 100 WLM, based on the highest (3.2/1.22 = 2.62) (Hunter et al. 2013) and lowest (3.2/0.99 = 3.23) (L'Abbé et al. 1991) bias factors.

## Discussion

The joint cohort analysis of male uranium workers confirms a statistically significant linear exposure–response relationship between low cumulative radon exposures and lung cancer mortality (ERR/WLM = 0.022; 95% CI 0.013–0.034) based on 408 lung cancer deaths and 394,236 person-years of follow-up from 1953 to 1999. The exposure–response relationship was statistically significant and consistent across the three cohorts.

Our risk estimates and 95% confidence intervals are comparable to Czech, French, and German studies of uranium miners based on measured exposures, cohorts first employed after radiation protection measures were in place, and/or low cumulative radon exposures (Supplementary Table 3) (Tomasek et al. 2008; Rage et al. 2015; Kreuzer et al. 2015, 2018; Hunter et al. 2013; Leuraud et al. 2011; Vacquier et al. 2011). Our results were higher than recent Ontario and French cohort studies (Navaranjan et al. 2016; Rage et al. 2018) with > 50 years of follow-up and low cumulative radon exposures. Their lower risk estimates may reflect greater uncertainty in early estimated or extrapolated radon exposures which may underestimate radon risk.

We found a statistically significant monotonic increase in the relative risk of lung cancer mortality with increasing cumulative radon exposure for the joint cohort categorical analysis (*P* < 0.001). Workers with cumulative radon exposures of 80–100 WLM had a relative risk (RR 2.32; 95% CI 1.45–3.76) compared to the reference exposure category of 0.0 WLM (Fig. 1; Table 6).

An important result of this analysis is the apparent lack of an effect below 10 WLM cumulative radon exposures, where the risk estimates were around one. The results at this exposure range are imprecise, but may suggest a non-linear relationship. Although the upper confidence limits are compatible with an increased risk, predicted by a linear non-threshold model, the lower confidence limits are equally compatible with a reduced risk at low exposures model (i.e., hormesis). However, a conclusion of no effect or any effect is not possible because of very low statistical power at these exposures. Larger pooled studies would be useful to provide some answers.

Finally, a statistically significant decrease in the relative risk of lung cancer mortality with increasing time since exposure and a non-statistically significant decrease in risk with attained age were found. No exposure rate effect was found, consistent with several other studies of lung cancer risk at low radon exposures (National Research Council 1999; Tomasek et al. 2008; Lane et al. 2010; Rage et al. 2015; Kreuzer et al. 2018; Walsh et al. 2010; Hunter et al. 2013).

Information on tobacco smoking, the main risk factor for lung cancer, was not measured in the three cohorts being analyzed. Both sensitivity analyses suggested that a statistically significant linear radon–lung cancer relationship persisted after controlling for tobacco smoking under reasonable smoking scenarios. The first sensitivity analysis for unmeasured tobacco smoking (Steenland and Greenland 2004) observed an unadjusted RR of 2.07 at 50–99 WLM and an adjusted RR of 2.26 (range 1.88–2.33) using external smoking prevalence and tobacco–lung cancer relative risks (Hunter et al. 2013; Gandini et al. 2008). The second sensitivity analysis observed an unadjusted RR of 3.20 at 100 WLM and a range of adjusted RRs from 2.62 to 3.23 at 100 WLM using nested case–control studies and the 1960 + sub-cohort of  German uranium miners (Kreuzer et al. 2018; Hunter et al. 2013; Leuraud et al. 2007, 2011; Schnelzer et al. 2010; Tomasek 2011, 2013; L’Abbé et al. 1991).

### Strengths and limitations

The main strengths of this study were the large sample size, the relatively good quality, measured radon exposure data during the study periods, and good long-term cohort mortality ascertainment.

The joint cohort’s sample size substantially increased the statistical power of the individual cohorts (Tomasek et al. 2008; Lane et al. 2010; Vacquier et al. 2011; Rage et al. 2012). Higher and more precise risk estimates were found in this joint analysis than in the previous analyses of low radon exposures that were based on earlier time periods and included estimated or extrapolated radon exposure estimates (National Research Council 1999).

Harmonizing the cohorts by time periods of radiation protection measures, high number and quality of ambient radon measurements, and individual monitoring through PADs substantially increased the likelihood that radon progeny exposure measurements were of high quality (Tomasek et al. 2008; Lane et al. 2010). However, measurement error may still exist (Stayner et al. 2007). Substantial reductions in radon exposures from means of > 20 to < 5 WLM/year occurred during the time periods under study, largely due to mechanical ventilation systems and regulatory exposure limits (Tomasek et al. 2008; Lane et al. 2010). The past time periods of low mean annual radon exposures reflect modern occupational exposures although the exposures were still higher than current mean annual exposures (< 0.25 WLM/year from 2005 to 2015) (National Dose Registry, special tabulations, 2016-11-15).

The joint analysis had long-term and high-quality mortality follow-up of uranium miners to 1999. The Czech and Beaverlodge cohorts had almost complete ascertainment of lung cancer mortality. Histology was available for at least 80% of lung cancers in the Czech cohort. Of those who died; only 0.4% of the Czech cohort had missing causes of death (Tomasek et al. 2008). Causes of death were obtained for ~ 89% in the Eldorado cohort (1950–1999) (Zablotska et al. 2013). This percentage was likely greater for Beaverlodge workers employed after 1965 due to improvements in the quality of the national mortality database over time and the use of the SIN which substantially improved record linkage (CNSC 2004). Although the French national mortality database did not exist before 1968; only ~ 3% of overall miners had missing causes of death. Correcting for missing causes of death did not have a large impact on the exposure–response relationship, because most French workers were still alive in 1968. Thus, the number of lung cancer deaths before 1968 was small (Tomasek et al. 2008; Laurier et al. 2004).

The main limitation of this study was that only grouped person-year data, not individual data, were available for this analysis. Thus, analytic decisions such as choice of categorical variable cutoffs were limited. For time-varying factors, workers contributed to the appropriate category as time progressed. All workers, regardless of their final cumulative exposures, contributed person-years to the exposure data set. However, deaths would only reflect those of workers with < 100 WLM (or < 50 WLM, ≤ 5.0 WL), since any worker with a greater exposure would have died at a higher exposure level. Alternative analytic methods would have been possible if individual-level data were available. In general, grouped and ungrouped data provide equivalent results when modelled identically (Richardson et al. 2004; Loomis et al. 2005).

Had individual-level data been available, only those individuals with lifetime exposure < 100 WLM would have been analyzed. However, because only grouped data were available, the decision to exclude person-year strata at > 100 WLM from the primary analysis could have introduced a bias through censoring follow-up. Thus, individuals’ earlier and lower exposures might have causally affected the risk of lung cancer death, despite that they accumulated more exposure and eventually developed and died from lung cancer at a later time. However, an alternative approach of not excluding person-year strata at > 100 WLM and instead only reporting excess relative risks estimates in the range of 0–100 WLM would have potentially allowed bias from measurement error at higher levels of exposure, which were predominately accumulated in the earlier time periods. In fact, the excess relative risk was estimated when strata at < 100 WLM (Table 5, ERR/WLM = 0.022; 95% CI 0.013–0.034) was similar to that estimated without making this exclusion (ERR/WLM = 0.020; 95% CI 0.015–0.027).

We addressed potential healthy worker survivor bias (Buckley et al. 2015; Bjor et al. 2015; Picciotto and Hertz-Picciotto 2015) several ways. Radon exposures were lagged by 5 years to address any changes in a worker’s exposure due to lung cancer. Short-term workers were excluded, since they have higher mortality rates than long-term workers unrelated to occupational radon exposures (Buckley et al. 2015; Bjor et al. 2015). Although short-term Beaverlodge workers (< 6 months) did not have higher crude mortality rates, they had unique characteristics: higher proportion of person-years with age at risk 19–29 years (22.7% versus 4.8%) and cumulative exposure < 5 WLM (87.9% versus 45.3%) compared to long-term workers (≥ 6 months). For these reasons, we excluded them to be consistent with the Czech and French cohorts. Workers with less strenuous jobs (open pit miners, mill workers), independent of duration of work, were included, since we found no evidence that they were less healthy than underground miners (Lane et al. 2010; Vacquier et al. 2009). Workers with 0.0 WLM were included as the reference population, since they were not systematically different for those with higher exposures and all job types had some workers with 0.0 WLM.

The start of follow-up began after either 1 or 4 years of employment in the Czech and French cohorts. Unfortunately, the exclusion of miners employed for < 6 months (74 deaths, 97,617 person-years) introduced immortal time bias in the Beaverlodge cohort, because it was not possible to exclude the first 6 months of at risk person-time from person-year tabulations of Beaverlodge workers employed for ≥ 6 months (195 deaths, 150,964 person-years). Start of follow-up began the first day of employment rather than after the first 6 months had passed. This overestimated the person-years of Beaverlodge workers by 6 months and, therefore, could have resulted in underestimation of the radon–lung cancer relationship. However, the potential healthy worker survivor bias of short-term workers likely would have had a more important impact than the immortal time bias. Workers employed < 6 months had a skewed person-year distribution of age at risk 16–29 years and low cumulative radon exposures (mean 9.95 WLM). This would have affected the harmonization of the Beaverlodge cohort with the Czech and French cohorts.

Only miners who were “incident hires”, who were first employed on or after 1953 or 1956, respectively, were included in the Czech and French cohorts. However, “prevalent” hires were introduced into the Beaverlodge cohort, since the use of person-year tables meant that workers who were first employed before 1965 were still retained, but their person-time contributions before 1965 were excluded. About 13% (37,265 person-years) of total person-time occurred from 1950 to 1964.

Sensitivity analyses of the Beaverlodge cohort suggested that risk estimates may be slightly overestimated, but there was not a big influence of prevalent hires on the exposure–response relationship. The impact of prevalent and incident hires on the potential healthy worker survivor bias, based on the workers’ date of hire before or after the start of follow-up, has been assessed in two recent occupational cohort studies (Costello et al. 2011; Applebaum et al. 2007). Despite the loss of statistical power and a restricted exposure range, decreasing the relative proportion of prevalent to incident hires reduced healthy worker bias, resulting in stronger evidence for a dose–response between occupational exposures and cancer mortality.

We used two different sensitivity analyses to assess the magnitude of the confounding effect of tobacco smoking. The first sensitivity analysis varied miners’ presumed smoking prevalence and the strength of the smoking–lung cancer mortality relationship to assess the sensitivity of the results under different plausible tobacco smoking scenarios. While our approach assumed that smoking-related risk estimates were generalizable to uranium miners, the meta-analysis (Gandini et al. 2008) included different populations and different smoking status definitions than those used in this study (Hunter et al. 2013). Smoking intensity may be a better measure than smoking status. Unfortunately, studies of radon-exposed miners (Villeneuve et al. 2007; Schubauer-Berigan et al. 2009) assessed the impact of smoking intensity only at > 100 WLM. We assumed that the effect of radon was constant across levels of smoking status; however, the joint effect of tobacco smoking and radon is between an additive and multiplicative interaction (National Research Council 1999; Kreuzer et al. 2018; Hunter et al. 2013; Leuraud et al. 2007, 2011; Schubauer-Berigan et al. 2009; Tomasek 2011). Nested case–control studies and the 1960 + sub-cohort of German uranium miners suggest that estimates of parameters in relative risk models are relatively close between estimates of parameters when smoking is adjusted for and when smoking is ignored. Likewise, these estimates correspond to the risk in smokers (majority of cases). The overall risk (in smokers + never smokers) is somewhat higher, because risk coefficients in never smokers are higher by a factor of ~ 2–3 (National Research Council 1999; Kreuzer et al. 2018).

The second sensitivity analysis likely better reflects the bias due to unmeasured smoking, since it was derived from nested case–control studies and a sub-cohort of uranium miners similar to those in our study (Kreuzer et al. 2018; Hunter et al. 2013; Leuraud et al. 2007, 2011; Schnelzer et al. 2010; Tomasek 2011, 2013; L’Abbé et al. 1991). The European joint nested case–control study at < 100 WLM (Hunter et al. 2013) and restricted to later time periods (Leuraud et al. 2011) are likely most reflective of our joint cohort analysis, since they include the French and Czech cohorts, low radon exposures and time periods of quality exposures. The larger size of the joint nested case–control study (Hunter et al. 2013; Leuraud et al. 2011) and the 1960 + sub-cohort of the German cohort study (Kreuzer et al. 2018), compared to the cohort-specific nested case–control studies (Schnelzer et al. 2010; Tomasek 2011, 2013; L’Abbé et al. 1991; Leuraud et al. 2007) provided more statistical power to assess the risk of lung cancer mortality at low radon exposures, adjusting for tobacco smoking. Nonetheless, measures of tobacco smoking status were crude in the sensitivity analysis, so had implications for residual confounding.

We assessed the impact of tobacco smoking on the radon–lung cancer relationship, because confounding can play a larger relative role when evaluating small effect sizes. Nonetheless, examples of substantial confounding are rare in studies of occupational exposures and lung cancer (Blair et al. 2007); tobacco-adjusted RRs are rarely appreciably different from unadjusted estimates. Our tobacco smoking sensitivity analyses support this outcome.

Gamma radiation, long-lived alpha radionuclides, residential radon, arsenic, silica, and diesel exhaust are human carcinogens (IARC 2012a, 2012b, 2013) and were reviewed as potential confounding factors for this study (Lane et al. 2010; Rage et al. 2012, 2015; Tomasek 2013; Walsh et al. 2010; Leuraud et al. 2011; Vacquier et al. 2011; Amabile et al. 2009). Many findings on other well-established human carcinogens indicate that confounding in occupational studies of lung cancer is rare and is not likely to be an explanation for positive study findings (Bruske-Hohlfeld et al. 2000; Lubin et al. 2000; Richiardi et al. 2005). If tobacco use does not confound lung cancer risks in occupational studies, it is even less likely that those more modest risk factors for lung cancer, with no known association with the occupational radon exposure of interest, would have a substantial effect (Blair et al. 2007).

### Implications

Today’s uranium miners are exposed to very low cumulative radon exposures. For example, the mean annual radon exposures of Canadian uranium miners ranged from 0.106 to 0.214 WLM from 2005 to 2015; the sum of 10 years average annual radon exposure was ~ 1.8 WLM (National Dose Registry, special tabulations, 2016-11-15). Although the same standards are applied worldwide for radiation protection, not all countries necessarily achieve the same low levels.

Joint analyses of existing cohorts of radon-exposed miners that are restricted to time periods of high quality, measured and low cumulative radon exposures, with long-term mortality and cancer incidence follow-up, may provide our best estimates of lung cancer risk at low cumulative radon exposures. These joint analyses are necessary to provide the statistical power to assess different aspects of this risk relationship. Precise quantification of the health risks at low cumulative radon exposures and the factors that confound and modify this risk are essential to provide objective scientific information to support radiation protection (ICRP 2017).

## Conclusion

Our analysis adds precise risk estimates of the risk of lung cancer mortality at low cumulative radon exposures < 100 WLM. It is based on three cohorts of uranium miners who were first employed after radon mitigation measures were in place and who had high-quality radon exposure measurements and long-term mortality follow-up. The study has important occupational and public health implications. The analyses of joint cohorts of uranium miners with high-quality and low cumulative exposures and extended mortality follow-up are important for ongoing assessment of occupational radon–lung cancer mortality risk.

## Electronic supplementary material

Below is the link to the electronic supplementary material.

Supplementary material 1 (PDF 247 KB)

## References

[CR1] Amabile JC, Leuraud K, Vacquier B, Caer-Lorho S, Acker A, Laurier D (2009). Multifactorial study of the risk of lung cancer among French uranium miners: radon, smoking and silicosis. Health Phys.

[CR2] Applebaum KM, Malloy EJ, Eisen EA (2007). Reducing healthy worker survivor bias by restricting date of hire in a cohort study of Vermont granite workers. Occup Environ Med.

[CR3] Bjor O, Damber L, Jonsson H, Nilsson T (2015). A comparison between standard methods and structural nested modelling when bias from a healthy worker survivor effect is suspected: an iron-ore mining cohort study. Occup Environ Med.

[CR4] Blair A, Stewart P, Lubin JH, Forastiere F (2007). Methodological issues regarding confounding and exposure misclassification in epidemiological studies of occupational exposures. Am J Ind Med.

[CR5] Breslow NE, Day NE (1987). Statistical methods in cancer research. The design and analysis of cohort studies (IARC Scientific Publications No. 82).

[CR6] Bruske-Hohlfeld I, Mohner M, Pohlabeln H, Ahrens W, Bolm-Audorff U, Kreienbrock L (2000). Occupational lung cancer risk for men in Germany: results from a pooled control study. Am J Epidemiol.

[CR7] Buckley JP, Keil AP, McGrath LJ, Edwards JK (2015). Evolving methods for inference in the presence of healthy worker survivor bias. Epidemiology.

[CR8] CNSC (2003) Feasibility study: part II of the Saskatchewan Uranium Miners’ Cohort Study. RSP Report No. 0178. http://www.nuclearsafety.gc.ca/eng/resources/health/healthstudies/feasibility-study-saskatchewan-uranium-miners-cohort-study.cfm

[CR9] CNSC (2004) Summary report of the Eldorado nuclear cohort study: internal linkage, ‘alive’ follow-up, 1950–2000 mortality linkage, 1969–2000 cancer incidence linkage (prepared by Statistics Canada). RSP Report No. 0188

[CR10] Costello S, Friesen MC, Christiani DC, Eisen EA (2011). Metalworking fluids and malignant melanoma in autoworkers. Epidemiology.

[CR11] Gandini S, Botteri E, Iodice S, Bonio M, Lowenfels AB, Maisonneuve P (2008). Tobacco smoking and cancer: a meta-analysis. Int J Cancer.

[CR12] Health Canada (2016) Report on occupational radiation exposures in Canada 2017. http://publications.gc.ca/site/archiveearchived.html?url=http://publications.gc.ca/collections/collection_2018/sc-hc/H126-1-2017-eng.pdf

[CR13] Hunter N, Muirhead CR, Tomášek L, Kreuzer M, Laurier D, Leuraud K (2013). joint analysis of three European nested control studies of lung cancer among radon exposed miners: exposure restricted to below 300 WLM. Health Phys.

[CR14] IARC (2012). Arsenic, metals, fibres and dusts. A review of human carcinogens. IARC monographs on the evaluation of carcinogenic risks to humans.

[CR15] IARC (2012). Radiation. A review of human carcinogens. IARC monographs on the evaluation of carcinogenic risks to humans.

[CR16] IARC (2012). Personal habits and indoor combustions. A review of human carcinogens. IARC monographs on the evaluation of carcinogenic risks to humans.

[CR17] IARC (2013) Diesel and gasoline engine exhausts and some nitroarenes. IARC monographs on the evaluation of carcinogenic risks to humans, vol 105. International Agency for Research on Cancer, Lyon. http://publications.iarc.fr/Book-And-Report-Series/Iarc-Monographs-On-The-Identification-Of-Carcinogenic-Hazards-To-Humans/Diesel-And-Gasoline-Engine-Exhausts-And-Some-Nitroarenes-2013

[CR18] ICRP (2010). Lung cancer risk from radon and progeny and statement on radon. Ann ICRP.

[CR19] ICRP (2017) Occupational intakes of radionuclides: part 3. Ann ICRP 46(3,4) 10.1177/0146645317734963 29380630

[CR20] Kreuzer M, Fenske N, Schnelzer M, Walsh L (2015). Lung cancer risk at low radon exposure rates in German uranium miners. Br J Cancer.

[CR21] Kreuzer M, Sobotzki C, Schnelzer M, Fenske N (2018). Factors modifying the radon-related lung cancer risk at low exposures and exposure rates among german uranium miners. Radiat Res.

[CR22] L’Abbé KA, Howe GR, Burch JD, Miller AB, Abbatt J, Band P (1991). Radon exposure, cigarette smoking, and other mining experience in the Beaverlodge uranium miners cohort. Health Phys.

[CR23] Lane RS, Frost SE, Howe GR, Zablotska LB (2010). Mortality (1950–1999) and cancer incidence (1969–1999) in the cohort of Eldorado uranium workers. Radiat Res.

[CR24] Laurier D, Tirmarche M, Mitton N, Valenty M, Richard P, Poveda S (2004). An update of cancer mortality among the French cohort of uranium miners: extended follow-up and new source of data for causes of death. Eur J Epidemiol.

[CR25] Leuraud K, Billon S, Bergot D, Tirmarche M, Caer S, Quesne B (2007). Lung cancer risk associated to exposure to radon and smoking in a control study of French uranium miners. Health Phys.

[CR26] Leuraud K, Schnelzer M, Tomášek L, Hunter N, Tirmarche M, Grosche B (2011). Radon, smoking and lung cancer risk: results of a joint analysis of three European control studies among uranium miners. Radiat Res.

[CR27] Loomis D, Richardson DB, Elliott L (2005). Poisson regression analysis of ungrouped data. Occup Environ Med.

[CR28] Lubin JH, Tomasek L, Edling C, Hornung RW, Howe G, Kunz E (1997). Estimating lung cancer mortality from residential radon using data for low exposures of miners. Radiat Res.

[CR29] Lubin JH, Pottern LM, Stone BJ, Fraumeni JF (2000). Respiratory cancer in a cohort of copper smelter workers: results from more than 50 years of follow-up. Am J Epidemiol.

[CR30] National Research Council (1999) Health effects of exposure to radon: BEIR VI. Committee on health risks of exposure to radon (BEIR VI). Board on radiation effects research commission on life sciences. National Academy Press, Washington, D.C.

[CR31] Navaranjan G, Berriault C, Do M, Villeneuve PJ, Demers PA (2016). Cancer incidence and mortality from exposure to radon progeny among Ontario uranium miners. Occup Environ Med.

[CR32] Picciotto S, Hertz-Picciotto I (2015). Commentary: healthy worker survivor bias: a still-evolving concept. Epidemiology.

[CR33] Preston D (1996) Statistical Methods in Studies of Radiation Risk. In: SAS conference proceedings. South East SAS Users Group (SESUG) 1996 Proceedings; October 13–15, 1996; Lex Jansen and SAS Institute Inc.

[CR34] Preston DL, Shilnikova NS (2017) EPICURE Version 2 User Guide. http://epicure.risksciences.com. Accessed 30 Oct 2017

[CR36] Rage E, Vacquier B, Blanchardon E, Allodji RS, Marsh JW, Caer-Lorho S (2012). Risk of lung cancer mortality in relation to lung doses among French uranium miners: follow-up 1956–1999. Radiat Res.

[CR37] Rage E, Caer-Lorho S, Drubay D, Ancelet S, Laroche P, Laurier D (2015). Mortality analyses in the updated French cohort of uranium miners (1946–2007). Int Arch Occup Environ Health.

[CR38] Rage E, Caer-Lorho S, Laurier D (2018). Low radon exposure and mortality among Jouac uranium miners: an update of the French cohort (1946–2007). J Radiol Prot.

[CR39] Richardson D, Wing S, Steenland K, McKelvey W (2004). Time-related aspects of the healthy worker survivor effect. Ann Epidemiol.

[CR40] Richardson DB, Cole SR, Langholz B (2012). Regression models for the effects of exposure rate and cumulative exposure. Epidemiology.

[CR41] Richiardi L, Forastiere F, Boffetta P, Simonato L, Merletti F (2005). Effect of different approaches to treatment of smoking as a potential confounder in a control study on occupational exposures. Occup Environ Med.

[CR42] Schnelzer M, Hammer GP, Kreuzer M, Tschense A, Grosche B (2010). Accounting for smoking in the radon-related lung cancer risk among German uranium miners: results of a nested control study. Health Phys.

[CR43] Schubauer-Berigan MK, Daniels RD, Pinkerton LE (2009). Radon exposure and mortality among white and American Indian uranium miners: an update of the Colorado Plateau cohort. Am J Epidemiol.

[CR44] Statistics Canada (2013). An examination of the NAACCR method of assessing completeness of case ascertainment using the Canadian Cancer Registry (by D Zakaria). Health Rep.

[CR45] Stayner L, Vrijheid M, Cardis E, Stram DO, Deltour I, Gilbert SJ (2007). A Monte Carlo maximum likelihood method for estimating uncertainty arising from shared errors in exposures in epidemiological studies of nuclear workers. Radiat Res.

[CR46] Steenland K, Greenland S (2004). Monte Carlo sensitivity analysis and Bayesian analysis of smoking as an unmeasured confounder in a study of silica and lung cancer. Am J Epidemiol.

[CR47] Tirmarche M, Laurier D, Bochicchio F, Cardis E, Binks K, Hofmann W et al (2010) Alpha Risk—final scientific report—Version 2.0. Quantification of cancer and non-cancer risks associated with multiple chronic radiation exposures: epidemiological studies, organ dose calculation and risk assessment. Project No. 516483

[CR48] Tomasek L (2011). Interaction of radon and smoking among Czech uranium miners. Radiat Prot Dosimetry.

[CR49] Tomasek L (2012). Lung cancer mortality among Czech uranium miners-60 years since exposure. J Radiol Prot.

[CR50] Tomasek L (2013). Lung cancer risk from occupational and environmental radon and role of smoking in two Czech nested control studies. Int J Environ Res Public Health.

[CR51] Tomasek L, Rogel A, Tirmarche M, Mitton N, Laurier D (2008). Lung cancer in French and Czech uranium miners: Radon-associated risk at low exposure rates and modifying effects of time since exposure and age at exposure. Radiat Res.

[CR52] Vacquier B, Rogel A, Leuraud K, Caer S, Acker A, Laurier D (2009). Radon-associated lung cancer risk among French uranium miners: modifying factors of the exposure-risk relationship. Radiat Environ Biophys.

[CR53] Vacquier B, Rage E, Leuraud K, Caer-Lorho S, Houot J, Acker A (2011). The influence of multiple types of occupational exposure to radon, gamma rays and long-lived radionuclides on mortality risk in the French “post-55” sub-cohort of uranium miners: 1956–1999. Radiat Res.

[CR54] Villeneuve PJ, Morrison HI, Lane R (2007). Radon and lung cancer risk: an extension of the mortality follow-up of the Newfoundland fluorspar cohort. Health Phys.

[CR55] Walsh L, Tschense A, Schnelzer M, Dufey F, Grosche B, Kreuzer M (2010). The influence of radon exposures on lung cancer mortality in German uranium miners, 1946–2003. Radiat Res.

[CR56] Walsh L, Grosche B, Schnelzer M, Tschense A, Sogl M, Kreuzer M (2015). A review of the results from the German Wismut uranium miners cohort. Radiat Prot Dosimetry.

[CR57] WHO (1998). International classification of diseases, ninth revision (ICD-9).

[CR58] Zablotska LB, Lane RS, Frost SE (2013). Mortality (1950–1999) and cancer incidence (1969–1999) of workers in the Port Hope cohort study exposed to a unique combination of radium, uranium and gamma-ray doses. BMJ Open.

