# Supplementary material for: Low radon exposures and lung cancer risk: joint analysis of the Czech, French, and Beaverlodge cohorts of uranium miners
Source: Int Arch Occup Environ Health. 2019 Feb 8;92(5):747–62. doi: 10.1007/s00420-019-01411-w (PMC6556158; doi:10.1007/s00420-019-01411-w)
Supplement: Supplementary file 1 — Supplementary material 1 (PDF 247 KB) [file 420_2019_1411_MOESM1_ESM.pdf]

Supplementary Table 1 Prevalence of tobacco smoking among controls, by radon exposure categories in a European joint nested case-control study *(16)***(Nezahat Hunter, special tabulations, 2016-10-17)**

Cumulative radon exposure (WLM)	Never-smoker N (%)	Ex-smoker ≥ 10 years N (%)	Current and Ex-smoker < 10 years N (%)
< 25	149 (20.4)	195 (26.7)	385 (52.8)
25–49	71 (31.1)	60 (26.3)	97 (42.5)
50–99	136 (30.9)	89 (20.2)	215 (48.9)

Table only provides smoking prevalence for exposure categories less than 100 WLM.
Tobacco smoking prevalence reflects the distribution in controls only.

Supplementary Table 2 Smoking unadjusted and smoking adjusted relative risks of lung cancer mortality at 100 WLM and bias ratios for various case-control studies

Nested case-control study	Smoking-unadjusted RR at 100 WLM	Smoking-adjusted RR at 100 WLM	Bias ratio
Leuraud et al. 2011 *(17)*	2.00	1.80	1.11
Leuraud et al. 2011 *(17)* (CZ 1953–, FR & GR 1956–)	2.70	2.50	1.08
Hunter et al. 2013 *(16)* (< 300 WLM)	3.30	2.70	1.22
Hunter et al. 2013 *(16)* (< 100 WLM)	4.90	4.70	1.04
Tomasek et al. 2011 *(24)*	2.80	2.50	1.12
Tomasek et al. 2013 *(25)*	2.40	2.30	1.04
Leuraud et al. 2007 *(27)*	1.98	1.85	1.07
Schnelzer et al. 2010 *(23)*	1.25	1.23	1.02
L'Abbé et al. 1991 *(26)*	4.89	4.93	0.99
Kreuzer et al. 2018 *(11)* (smoking status available for 56% of 1960+ sub-cohort)	2.7	2.3	1.17

Cumulative radon exposures (WLM) were five-year lagged.
Nested case-control studies were matched on cohort, attained age and age at birth.
CZ: Czech; FR: French; GR: German; BL: Beaverlodge.
The bias factor due to smoking difference in each nested case-control study equals the $RR_{unadjusted}$ / $RR_{adjusted}$ at 100 WLM within the specific nested case-control study.

Supplementary Table 3 Summary of lung cancer mortality (ERR/100 WLM) by cumulative radon exposure at low exposures or exposure rates from studies of uranium miners

Reference	No. of cohorts	Included	Lung cancer deaths (cases/controls)	Person-years	ERR/WLM	95% CI
Lubin et al. 1997 *(5)*	11	< 100 WLM	562	564,772	0.008	0.003–0.014
		< 50 WLM	353	453,604	0.012	0.002–0.025
Tomasek et al. 2008 *(4)* Cz 1952–95, Fr 1946–94	2	All Measured exposures	574	248,782	0.027	0.017–0.043
Leuraud et al. 2011 *(17)* Late sample: Cz 1953–99, Fr 1956–94 & Gr 1956–98	3	All	(418/1234)		0.017	0.009–0.029
Hunter et al. 2013 *(16)* Cz 1948–99, Fr 1946–94 & Gr 1946–98	3	All	(1046/2492)		0.008	0.004–0.014
		< 300 WLM	(742/2088)		0.017	0.009–0.035
Vacquier et al. 2011(*18*) Fr post-1955: 1956–99	1	All External regression	66	89,405	0.0210	0.0058–0.0488
		All Internal regression	66	89,405	0.0212	0.0053–0.0528
Rage et al. 2015 *(8)* Fr post-1955: 1956–07	1	All External regression	94	110,548	0.023	0.009–0.0492
		All Internal regression	94	110,548	0.024	0.009–0.0514
Kreuzer et al. 2015 *(13)* Gr 1960–08	1	All	334	846,809	0.013	0.007–0.021
		< 100 WLM	306	834,090	0.016	0.008–0.028
		< 50 WLM	243	794,923	0.013	0.001–0.029
Kreuzer et al. 2018 *(11)* Gr 1946–13:	1	<100 WLM Measured exposures	1,254	1,620,190	0.016	0.008–0.028
Gr 1960–13:		Measured exposures	495	956,776	0.017	0.007–0.032
Rage et al. 2018 *(12)* Fr 1946–07	1	All	211	186,994	0.0073	0.0032–0.0133
Navaranjan et al. 2016 *(10)* Ont. 1954–07	1	All	1,230	884,828	0.0064	0.0043–0.0085

CI confidence interval

The studies are based on either restricted to low cumulative exposures, or time periods when mechanical ventilation, routine radon monitoring and high quality exposure measurements were in place.
